# Combined Approaches for Drug Design Points the Way to Novel Proline Racemase Inhibitor Candidates to Fight Chagas’ Disease

**DOI:** 10.1371/journal.pone.0060955

**Published:** 2013-04-16

**Authors:** Armand Berneman, Lory Montout, Sophie Goyard, Nathalie Chamond, Alain Cosson, Simon d’Archivio, Nicolas Gouault, Philippe Uriac, Arnaud Blondel, Paola Minoprio

**Affiliations:** 1 Laboratoire des Processus Infectieux à Trypanosomatidés, Département Infection et Epidémiologie, Institut Pasteur, Paris, France; 2 Unité de Bioinformatique Structurale, CNRS-UMR 3528, Département de Biologie Structurale et Chimie, Institut Pasteur, Paris, France; 3 Equipe Produits Naturels, Synthèses et Chimie Médicinale, UMR 6226 Sciences Chimiques de Rennes, Université de Rennes 1, Rennes, France; University of Edinburgh, United Kingdom

## Abstract

Chagas’ disease is caused by *Trypanosoma cruzi*, a protozoan transmitted to humans by blood-feeding insects, blood transfusion or congenitally. Previous research led us to discover a parasite proline racemase (*Tc*PRAC) and to establish its validity as a target for the design of new chemotherapies against the disease, including its chronic form. A known inhibitor of proline racemases, 2-pyrrolecarboxylic acid (PYC), is water-insoluble. We synthesized soluble pyrazole derivatives, but they proved weak or inactive *Tc*PRAC inhibitors. *Tc*PRAC catalytic site is too small and constrained when bound to PYC to allow efficient search for new inhibitors by virtual screening. Forty-nine intermediate conformations between the opened enzyme structure and the closed liganded one were built by calculating a transition path with a method we developed. A wider range of chemical compounds could dock in the partially opened intermediate active site models *in silico*. Four models were selected for known substrates and weak inhibitors could dock in them and were used to screen chemical libraries. Two identified soluble compounds, *(E)-*4-oxopent-2-enoic acid (OxoPA) and its derivative *(E)-*5-bromo-4-oxopent-2-enoic acid (Br-OxoPA), are irreversible competitive inhibitors that presented stronger activity than PYC on *Tc*PRAC. We show here that increasing doses of OxoPA and Br-OxoPA hamper *T. cruzi* intracellular differentiation and fate in mammalian host cells. Our data confirm that through to their binding mode, these molecules are interesting and promising as lead compounds for the development of chemotherapies against diseases where active proline racemases play essential roles.

## Introduction

Chagas’ disease is one of the main causes of death by heart failure in South and Central America. With at least 12 million people infected and 100 million at risk, this is both a major health concern and a socioeconomic problem in Latin America. As a “Most Neglected Disease”, it is the third largest health burden after malaria and schistosomiasis [1], [2]. Of the affected adult population, 10% will die from this chronic disease, which is incurable and fatal in children aged less than two years. No vaccine is available and current therapies are only effective in the acute phases of the disease, while their success in chronic phases remains a matter of debate. Recently, French Guyana experienced an outbreak of the disease and this *per se* had a substantial impact on European authorities that implemented eligibility criteria for donors of blood, blood components, cells and tissues [3]. Recent increases in congenital transmission, blood transfusion and transplantation have drawn the attention of Public Health actors both in Europe and the USA [4]–[6].

Until recently, only two drugs were available to treat infected patients: Nifurtimox (3-methyl-*N-*[(*E*)-(5-nitrofuran-2-yl)methylidene]thiomorpholin-4-amine-1,1-dioxide) and Benznidazole, a nitroimidazole derivative (*N*-benzyl-2-(2-nitro-1*H*-imidazol-1-yl)acetamide) [7]. Because of the side effects it causes, such as neurological and gastrointestinal disorders the use of Nifurtimox has been limited in favor of Benznidazole. Chagas disease treatment, Benznidazole is today the only nitro-bearing medicine [8]. Although its mechanism of action is not fully elucidated, the drug is known to interfere with the redox cycles of intracellular and extracellular parasite forms [9], [10]. Several toxic but reversible effects have been described such as general allergic dermopathy, peripheral polyneuropathy, leucopenia, anorexia and convulsions [8]. Major complications include agranulocytosis and thrombopenic purple. In addition, Benznidazole is mostly effective in the early stages while most individuals afflicted with Chagas disease are diagnosed once the chronic phases is already installed. Consequently, although eradication of the parasite’s insect vector reduce its incidence in humans, new and more efficient treatments are needed and are considered a priority [11], [12].

Our previous research led to the identification and characterization of several isoforms of *T. cruzi* proline racemases (*Tc*PRAC) [13], [14]. Secreted and intracellular forms of the enzyme were detected at all stages of the parasite life cycle. The secreted form was shown to be a B-cell mitogen, which contributes to parasite avoidance of the host immune system, and to its persistence [13]. *Tc*PRAC is a promising target for the development of a new therapy against Chagas disease since parasites are no longer viable when *PRAC* genes are knocked down or more virulent if PRAC genes are over expressed [15]. Moreover, our current results using the 2-pyrrolecarboxylic acid (PYC), the competitive (water insoluble) inhibitor of PRAC [16], indicate that the infection of host cells *in vitro* reduces in a clearly dose-dependent manner when PYC is added at parasite-host cell interaction step *in vitro*. Lower mean numbers of parasites *per* cell are also noted [17]. Interestingly, we demonstrated that PYC binding closes the catalytic crevice and impacts on the overall structure of the enzyme, precluding its interaction with B-cells.

Here, we describe our approach to identifying new and more effective *Tc*PRAC inhibitors, which could be active in chronic phases of Chagas disease. First, we used classical medicinal chemistry approaches to improve PYC’s solubility and its affinity for the PRAC catalytic pocket. Several PYC analogues were synthesized and although they proved to be soluble in water they presented little activity toward PRAC. Two of the most soluble PYC derivatives, with a Chloro- or a Bromo- at C-4 position, weakly inhibited *Tc*PRAC. Next, since functional, biophysical and structural data were available for *Tc*PRAC [18], [19], virtual screening of chemical libraries to identify new types of inhibitors seemed to be an option. However, previous findings by crystallography and ITC calorimetry showed that PYC is completely buried within an extremely small and specific pocket in the “closed” ligand-bound structure of the enzyme [18]. Therefore, attempts to use pharmacophores designed by computational chemistry based on the crystallography of the complex “*Tc*PRAC/PYC” were unsuccessful because no ligand has been identified through the virtual screening of several databases of chemical compounds. Consequently, restricting our investigations to catalytic-site based models limited the virtual screening of chemical libraries to the identification of small and metabolite-like compounds. To overcome these restrictions, we built conformational transition paths between the closed (complexed with the inhibitor) and opened (inhibitor-free) shapes adopted by the *Tc*PRAC, which yielded a number of plausible intermediate structures of the catalytic site. Increasingly opened and wider active site models were used for the virtual screening of two accessible chemical libraries. Two of the selected products, OxoPA and its derivative, Br-OxoPA [20], were shown to be respectively 2- and 4-fold more potent than PYC in inhibiting *Tc*PRAC. Our results showed that OxoPA and Br-OxoPA have lower apparent K_i_ values than PYC and are irreversible competitive inhibitors. Finally, increasing doses of OxoPA and Br-OxoPA in DMSO caused greater decreases in mammalian host cell infection than those previously observed with PYC [17]. These findings confirmed these compounds as promising leads for the optimization and development of new therapies against diseases where proline racemases play an essential role.

## Materials and Methods

### Synthesis of Pyrazole Analogues

**Figure 1:**
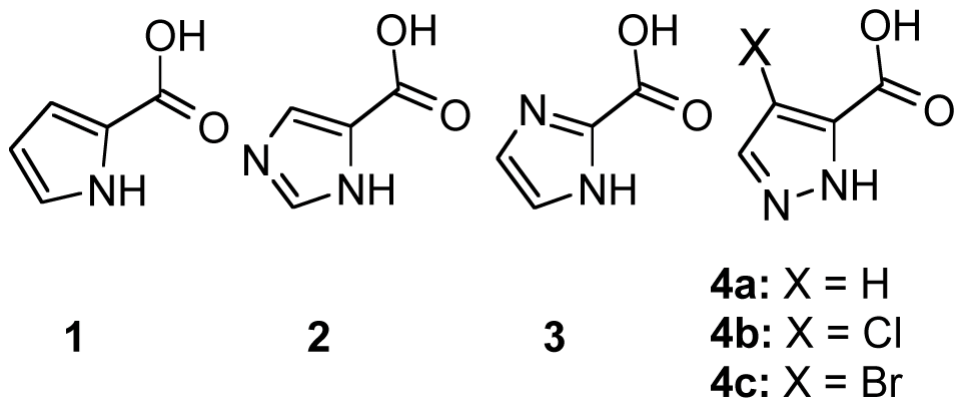
Structure of initial compounds and analogues. (1) PYC and its nitrogen-bearing analogues: (2) Imidazole-4-carboxylic acid, (3) 1-*H*-Imidazole-2-carboxylic acid and (4a) PZC, (4b) Cl-PZC and (4c) Br-PZC.

Figure 1 shows PYC (**1**), the two commercially available imidazole-bearing analogues (2) Imidazole-4-carboxylic acid and (**3**)1*H*-imidazole-2-carboxylic acid, and the pyrazole-bearing analogues **4a–c**. These were obtained from potassium permanganate oxidation of the corresponding 3-methylpyrazoles using previously described [21]–[23] and specific procedures [24]. Analogue **4a**, 1*H*-pyrazole-3-carboxylic acid is called PZC here. Analogues 4X-1*H*-pyrazole-3-carboxylic acids (X-PZC) for X = Cl (**4b**, Cl-PZC), X = Br (**4c**, Br-PZC) X = CH_2_CH_3_ (**5**, Et-PZC) and 4-Cl-1-methyl-pyrazole-3-carboxylic acid (**13**, M-Cl-PZC) bearing faint activity are reported in Table 1. Other 3-carboxylpyrazoles derivatives bearing larger substituted groups on carbons 4 and 5 were also prepared but were all devoid of effects in the racemization assays (not shown).

**Table 1:** PYC derivatives and their capacity to inhibit* *Tc*PRAC.

Compound, Name (Abbreviation and #)**	Solubility	Inhibition	Ki (µM)***
Pyrrole-2-carboxylic acid (PYC, 1)	Ethanol	**++++++**	6–10
Pyrazole-3-carboxylic acid (PZC, 4a)	Water	**+/−**	>2000
4-Chloro-pyrazole-3-carboxylic acid (Cl-PZC, 4b)	Water	**+**	300
4-Bromo-pyrazole-3-carboxylic acid (Br-PZC, 4c)	Water	**+**	1000
4-Ethyl-pyrazole-3-carboxylic acid (Et-PZC, 5)	Water	**+/−**	>2000
4-Chloro-5-methyl-pyrazole-3-carboxylic acid (M-Cl-PZC)	Ethanol	**+/−**	>2000

* As determined by polarimetry and DAAO methodologies (see Materials and Methods).

** See Supporting Table 1 for compound formula.

*** As determined by classical Michaelian approaches.

### Building a Transition Path between Open and Closed Forms of *Tc*PRAC and Virtual Screening

**Figure 2:**
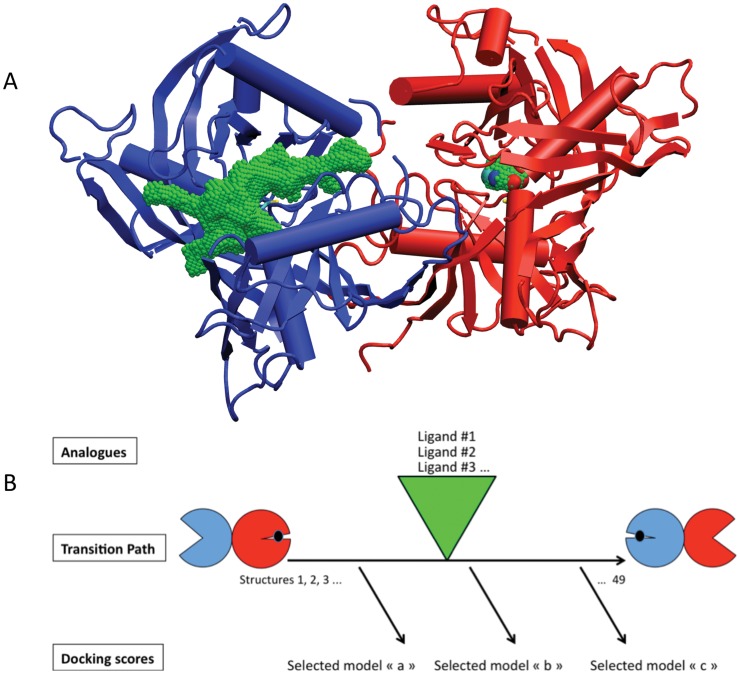
PYC-induced s*tructural rearrangement of Tc*PRAC prompted a virtual screening strategy. (A) In the crystal structure (1W62.PDB), the substrate-binding cavity of the “closed” protomer, shown as red ribbons and cylinders, is completely buried, as illustrated by the small green volume surrounding PYC whose nitrogen, oxygen and carbon appear as blue, red and cyan small spheres, respectively. In the absence of PYC, the *Tc*PRAC protomer has a more relaxed, open structure, shown as blue ribbons and cylinders, with the void volume of the active site accessible from the bulk solvent shown in green. (B) Schematic overview of the virtual screening strategy. The 3D structure of the hemi saturated complex shown in A is represented by circles of the same color on the left. It was used to build the symmetric form of the complex as depicted by the circles on the right. Molecular mechanics and molecular dynamics were used to model *Tc*PRAC motion (horizontal arrow) triggered by inhibitor binding. Known (weak) inhibitors derived from PYC were used to select intermediate conformations on the basis of their docking scores.

The structure of hemi-bounded TcPRAC (1W62 hemi complex PDB) is asymmetric and shows one closed subunit binding PYC (chain A), and one unbound opened subunit (chain B) [18]. By swapping the chain labels and reorientation on the original structure we built an inverted structure where chain A is opened and chain B is closed (see Figure 2A). Internal coordinates and Cartesian coordinates of the two extreme structures were compared to remove unnecessary symmetric side chain flips between the two structures. Then, we built a transition path connecting the two structures, hence modeling opening of chain A and closing of chain B when moving from the original structure to the inverted one (see schematic view in Figure 2B). For that, we used the “Path Optimization and Exploration” approach (POE) described in ref. [25]. This approach iteratively uses the Conjugated Peak Refinement (CPR) [26] method of the CHARMM program [27] to refine the path, explore possible shorter local paths and reassembles them to reconstruct the whole path. The transition path was built on all atomic degrees of freedom (here 19488), as a curved trajectory formed by a series of N (here 49 at the end of the procedure) ordered and low energy intermediate states (X_i_, i in [1,.,N]) avoiding energetic barriers. Following CPR construct, it ensures the absence of “hidden” barriers by probing the energy of structures along a straight line between consecutive intermediates of the path ([X_i_,X_i+1_], with i in [1,.,N−1]), and checks that it is lower than a given (low) threshold (E[(1–λ)X_i_+λX_i+1_]< E_max_, λ in [0,1]). To facilitate the first iteration, the procedure was initiated with a Molecular Dynamics (MD) trajectory starting from the original structure and drawn towards the inverted structure by a linear constraint of 0.5 kcal/mol/Å/atom. MD was 200,000 steps long recorded every 100 steps with a Langevin thermostat at 300 K and a friction coefficient of 100 ps^−1^ to remove excess heat. The solvent is modeled implicitly for CPR is an optimization method and, thus requires instantaneous solvent equilibration at room temperature while the structure energy is being minimized, which corresponds to a low temperature. The analytical continuum electrostatics (ACE2) potential [28] was used with default dielectric constants (1 inside the protein and 80 outside) to avoid formation of artifactual cavities. POE includes various procedures above simple CPR to reduce the number of intermediates for the same level of optimization (all intermediate have energies lower than a given threshold, here −34000 kcal/mol). (i) Intermediate structures are systematically removed if any linear interpolations between remaining consecutive structures are still below an energy threshold (-34000 kcal/mol) in a procedure implemented as “backward reduction” [25]. (ii) Sub-paths are recalculated with CPR between intermediate X_i_ and X_j_, j−i>n_min_, at distance d_ij_<d_max_, when the latter are separated by a sequence intermediate X_i+1_,…X_j−1_ covering a length (l_i,j_ = d_i,i+1_+…+d_j−1,j_) that is K time larger than d_ij_. K, d_max_ and n_min_ are adjusted according to path refinement according to computer resources available (e.g. maintain the list of i,j pairs lower than 1000). Distance between X_i_ and X_j_, d_i,j_ is the RMS difference between respective coordinates expressed in Å. In early phases the path is rugged and K has to be set at high level, say 5, d_max_ low, say 1.5 Å, and n_min_ high, say 10 or 20. At late phases the path is smoother, the number of pairs fulfilling the early criteria decreases and K can be set to lower values, say 1.5, d_max_ higher, say 2–3 Å, and n_min_ lower say 5. A Recalculated sub-path replaces the original one when its maximum energy is lower or equal to that of the replaced sub-path and the number of intermediates it contains is lower.

**Figure 3:**
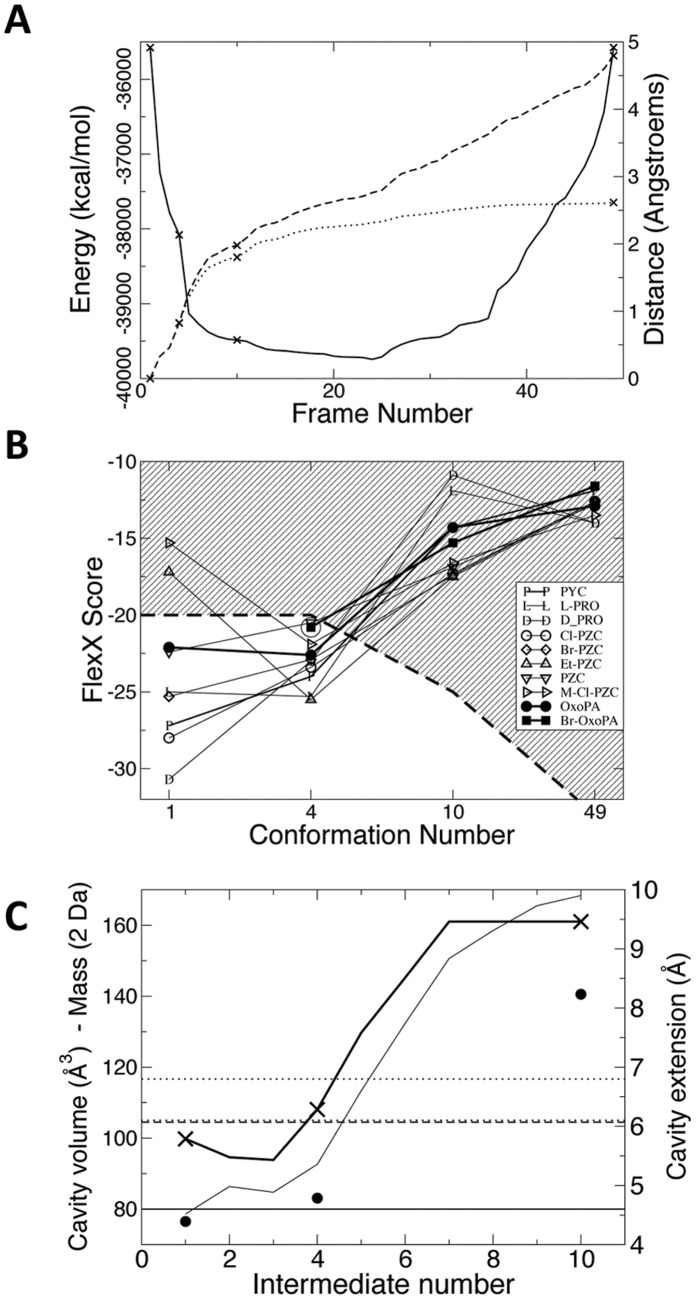
Transition path characteristics. (A) Energy profile and metric quantities for the *Tc*PRAC transition path. The energy profile (full line) shows that the intermediate states have low energy and do not present any energy barriers. Dotted and dashed lines show the distance from the first structure (d_1,i_) and the cumulative distance covered from the first structure (l_1,i_), respectively (RMS in Å, see Material and Methods). Little swerving was necessary to avoid the energy barriers. The points corresponding to the intermediate structures used in the screening are marked by crosses. (B) Scores of known ligands, synthesized analogues, and new inhibitors when docked in the selected binding site models. Br-OxoPA could not be docked in the crystallographic structure and its score in the fourth conformation is circled. The score threshold that was chosen in the subsequent virtual screening phase for ligand selection is indicated by a dashed line and the exclusion region is striped. Transition path characteristics. (A) Energy profile and metric quantities for the *Tc*PRAC transition path. The energy profile (full line) shows that the intermediate states have low energy and do not present any energy barriers. Dotted and dashed lines show the distance from the first structure (d_1,i_) and the cumulative distance covered from the first structure (l_1,i_), respectively (RMS in Å, see Material and Methods). Little swerving was necessary to avoid the energy barriers. The points corresponding to the intermediate structures used in the screening are marked by crosses. (B) Scores of known ligands, synthesized analogues, and new inhibitors when docked in the selected binding site models. Br-OxoPA could not be docked in the crystallographic structure and its score in the fourth conformation is circled. The score threshold that was chosen in the subsequent virtual screening phase for ligand selection is indicated by a dashed line and the exclusion region is striped. (C) Cavity volume and extension in transition path intermediates, and docked molecules properties. Volume and extension are calculated as explained in Material and Methods. The volume is displayed by the thin line curve. Cavity extension is displayed by the thick curve and crosses mark intermediates that were used for virtual screening. The extension of PYC is shown by the horizontal line, that of OxoPA in all-trans conformation is shown by the horizontal dashed line. The extension of BrOxoPA in all-trans conformation is given in dotted line for two extreme rotamers on the C4–C5 bond. The average molecular weight of the library compounds successfully docked in conformers 1, 4 and 10 is displayed by filled circles. For clarity, the average mass has been divided by 2 to fit the same scale as the cavity volume.

**Figure 4:**
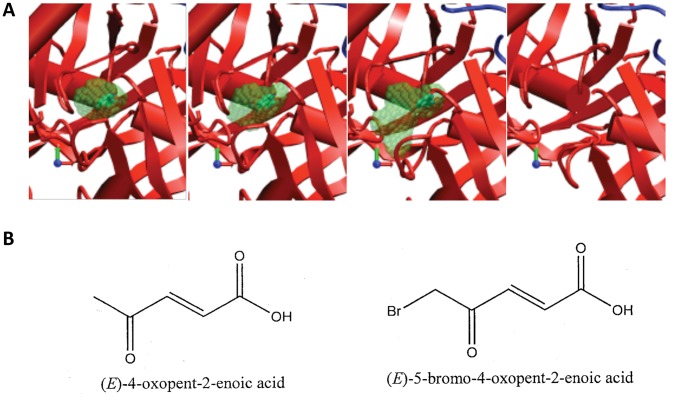
Selection of active site conformations for virtual screening. (A) Four of the 49 conformations defining the path were selected for virtual screening. Protein secondary structures are shown schematically as in Figure 2. Transparent green spheres show the enclosed void volume of the pocket, with the ligand inside the first three structures (opaque green). (B) Structure of the two identified novel inhibitors of *Tc*PRAC.

The volume of the cavity was calculated with a cavity search program developed in the laboratory and described in [29], but without outer sphere limit. Within the cavity, the points accessible to the center of the probe sphere are labeled (i.e. points excluded from the protein atoms when their canonical exclusion radii had been incremented by the solvent probe sphere radius). The cavity extension is calculated as the maximum distance between the latter points. Xmgrace was used to prepare the graphics of Figures 3A, 3B and 3C and Figure 4A [http://plasma-gate.weizmann.ac.il/Grace/]. Ligand sizes, l_max_, were measured on canonical conformers in the computer graphic program VMD, which was also used to prepare Figure 2A and Figure 4B [30].

Four intermediate structures from the transition path were selected using the docking scores of known ligands and weak inhibitors, and structural inspection. The FlexX 2.0.3 program was used [31] with the default scoring function as recommended by the provider for novel type of binding site and because the goal was to select different type of compounds than the known ones. Acidic amino acids and amines were deprotonated and protonated respectively. Dockings were performed twice with His 132 protonated on N_δ1_ and then, on N_ε2_. Doubly protonated His 132 gave similar geometries in redocking than His 132 singly protonated on N_δ1_ (pointing inwards the binding site) and was not used for screening. Compounds were assigned their best score obtained with the two His 132 protonation states. The 2D compound structure definition files (sdf) from two accessible chemical libraries: ChemDiv, Inc (San Diego, California, USA; 630,000 compounds at the time) and Chimiothèque Nationale (CNRS, France; 31,000 compounds at the time; library devoted to chemical diversity and not only to drugable compounds.) were processed with the CORINA program [32] to develop their 3D structure and add hydrogen atoms. No filters were applied to the library of compounds as their combination with the little size of the targeted pocket would most likely have obliterated any chance of finding discernible inhibitors. Ligands were selected if their FlexX score was below −20, −20, −25 and −33, respectively for transition path structures 1, 4, 10 and 49. Cutoff values were adjusted to trim selection to similar number of compounds as a compensation of the differences in volume and limit the number of candidates. (see Figure 3C; 219, 345, 385 and 430 compounds thus selected respectively). The compounds from the different screenings were combined (1199 unique compounds and 180 found more than once), clustered by chemical similarity (linear fingerprints) with an UPGMA classification and a 0.55 threshold in the ICM program [33], and the best compound in each cluster was selected.

### Preparation of Recombinant *Tc*PRAC

Recombinant *Trypanosoma cruzi* proline racemase (EC 5.1.1.4) was produced in *E. Coli* BL21 (DE3) (Invitrogen) and purified by immobilized metal affinity chromatography on nickel columns, as previously described [13].

### Racemization of L-Proline and Inhibition Assays

Optimum Proline racemization conditions for TcPRAC were determined using 10–300 mM L-Proline in 0.2 M NaOAc over a range of pH values, as described [13] and L- to D- proline conversion took place in 1.5 mL reaction. Concentrations of D-proline formed were determined by optical rotation of the solution at 365 nm in a 10 cm optical path cell, thermostated at 37°C, using a polarimeter (Perkin Elmer 241 MC). Assays were also performed into microtiter plates (100 µL), as follows: dilutions of L- Proline (40 mM to 2.5 mM) in 0.2 M sodium acetate pH 6.0 and 0.25 mM *Tc*PRAC were loaded into microtiter plate wells with 0–20 µM PYC or similar concentrations of potential inhibitors. Controls omitted *Tc*PRAC and/or PYC. The microtiter plates were incubated for 30 minutes at 37°C. The enzyme was inactivated by heating in a microwave oven for 15 s at 900 Watts and shifting the pH to 8.3 by adding 6.8 µL of 0.235 M sodium pyrophosphate. The presence of D-Proline was tested in 50 µL from each well using the D-amino oxidase - Horse Radish Peroxidase/OPD combined test [34]. Interference caused by the possible reactivity of amino acid L-forms with DAAOx was taken into account by using standards consisting of serial dilutions of an equimolar mixture of D- and L-proline ranging from 0 to 10 mM (10 mM total). For instance, DAAOx reacts with L-Proline 300 times slower than with D-Proline. Optical densities, OD_490 nm_ – OD_650 nm_ were recorded on a microtiter plate reader (Molecular Devices), and the signals from the blank control wells were deducted.

### Parasites, Cell Cultures and Cellular Extracts

Cell culture-derived trypomastigotes from *T. cruzi* CL Brener (clone F11-F5) were isolated from the supernatant of bulk cultures of green monkey Vero kidney cells previously infected with bloodstream trypomastigotes [35]. Known numbers of parasites, adherent infected cells and uninfected cell controls were lysed with 0.1 mL of 0.05% Tween 20 solution in sterile distilled water and the lysates were frozen. Vero cells were seeded in LabTek slides (5×10^4^ cells/well) in RPMI 1640 medium/5% FCS and kept at 37°C, 5% CO_2_. To test the effect of the inhibitors in the initial steps of the host-parasite interaction, cultures were infected for 17 h at 37°C at a 10∶1 parasite/cell ratio with or without increasing doses of freshly prepared dilutions (0–30 µM) of OxoPA, Br-OxoPA [20], or (10–1000 µM) of PYC, previously dissolved in DMSO. To test the effect of the inhibitors on the parasite intracellular cycle, cultures were infected at 37°C for 17 hours without inhibitors, washed three times to eliminate extracellular parasites then incubated for up to 48 hours with fresh medium containing different dilutions of the compounds. All cultures were then washed with PBS, fixed and stained with Giemsa. The number of infected host cells was recorded along with the number of parasites *per* infected cell in at least 400 host cells, in duplicate experiments. Results were expressed as the endocytic index (EI) resulting from the product of the percentage of infected cells and the mean number of parasites per infected cell [36]. Control cultures were incubated in medium alone or with equal DMSO concentrations.

### Capture ELISA

Flat-bottomed microtiter plates (Nunc, Denmark) were coated overnight at 4°C with rabbit anti-*T. cruzi* polyclonal antibodies and further blocked for 4 h at room temperature (RT) with 1% BSA, 0.05% Tween 20 in PBS. After washings, lysate samples from infected or control Vero cultures, or from *T. cruzi*, were then added and incubated at 4°C for 18 h. Plates were washed with PBS-Tween and incubated for 2 h at RT with polyclonal mouse anti-*T. cruzi* chronic serum, washed again and incubated for 1 h at RT with HRP-secondary antibodies. Reactions were revealed with 1 mg/mL OPD/0.05 M citrate/phosphate buffer, and appropriately stopped with 3 M HCl. Optical densities were determined in a spectrophotometer at 450 nm and 650 nm, and analyzed by Softmax Pro software.

## Results

### Two Synthesized PYC Analogues are New (but Weak) Inhibitors of Proline Racemase

We used conventional medicinal chemistry approaches in an attempt to improve the solubility of the proline racemase competitive inhibitor pyrrole carboxylic acid (PYC) and its affinity for the *Tc*PRAC catalytic pocket (Figure 1, Table 1 and Table S1). Primarily, several pyrazole analogues were synthesized [23], [24], [37], but while some showed better solubility in water by qualitative standards, none showed greater affinity for *Tc*PRAC than PYC (K_i_ = 6–10 µM). Two of the soluble PYC derivatives, namely 4-chloro-1*H*-pyrazole-3-carboxylic acid (Cl-PZC, **4b**) and 4-bromo-1*H*-pyrazole-3-carboxylic acid (Br-PZC, **4c**), weakly inhibited *Tc*PRAC (K_i_≈0.3 mM and 1 mM, respectively). Two other derivatives Ethyl-1*H*-pyrazole-3-carboxylic acid (Et-PZC, **5**) and 4-chloro-5-methyl-1*H*-pyrazole-3-carboxylic acid (M-Cl-PZC, **13**) seemed to exert weak inhibition (K_i_>2 mM). These results are in agreement with the failure of the classical docking approach. Hence, the synthesis of a transition state analogue, more potent than PYC, is certainly not the best way to find an efficient *Tc*PRAC inhibitor for Chagas disease treatment.

### Building Intermediate *Tc*PRAC Conformations to Broaden the Chemoinformatic Search for Inhibitors

The *Tc*PRAC catalytic site is small (the volume of a proline) and makes extremely specific contacts with the ligand [18]. Several pharmacophores were designed based on the features of the crystal PYC-bound to the catalytic site of *Tc*PRAC and used for the virtual screening of several chemical libraries. However, the search has identified only small, metabolite-like molecules, and failed to identify any new inhibitors (Minoprio, P. and Afshar, M., unpublished; see Table S2). To overcome this limitation, we built plausible intermediate structures to model the functional transition path of the *Tc*PRAC between its closed/liganded and its opened/unliganded forms [18] (see Figure 2A). Use of intermediate structures of the catalytic site broadened the search to larger and chemically more diverse inhibitor candidates.

Molecular mechanics methods detailed in Material and Methods section were used to infer the plausible conformational intermediate states adopted by *Tc*PRAC when switching between the two symmetric hemi-bound states found in the 1W62 PDB entry (Figure 2B). The transition proved to be relatively simple compared with other cases (e.g. [38], [25]) and the process converged after 3 full cycles of the procedure, described in Materials and Methods section. The resulting path consisted of 49 low-energy structures (or conformations) (see Figure 3A). The two subunits followed different transition paths, offering a larger number of possible intermediate conformations for the active site (96 = 49×2–2). Thus, the path is asymmetric and its length (l_1,49_) is 4.78 Å, to connect the two extreme conformations at 2.62 Å distance (d_1,49_) (see Figure 3A). The ratio of the length over the distance for the whole path (4.78/2.62 = 1.82) indicates that the transition path is moderately, but still significantly, curved (a linear transition would give a ratio of 1 and a hemi-circle, π/2).

Known ligands and analogues, PYC, L-PRO, D-PRO, Cl-PZC, Br-PZC, Et-PZC and M-Cl-PZC were docked in both catalytic sites on each of the 49 conformations using FlexX [30]. The pocket corresponding to Chain A in the 1W62 PDB entry gave the best results both in terms of docking score and RMSD in the case of PYC. Figure 3B gives the main scores obtained with the 4 selected docking models (i.e. 1, 4, 10 and 49). As the goal was to extend docking to other types of ligands, no attempt was made to optimize the docking of all those compounds. Conformation 4 was retained as it gave good scores for some ligands that did not score well in conformation 1 (closed catalytic site for chain A in the 1W62 PDB entry), especially for Et-PZC which is one of the largest known ligand and which had its best score in conformation 4. Conformation 10 gave favorable scores compared to neighboring conformations, and was the last “closed conformation” (subsequent conformations had opened access to bulk solvent) and showed an organized hydrogen bond network at the catalytic site, which was not the case for subsequent conformations. Hence, it was selected as it was expected to allow the capture of larger and more diverse ligands although the known ligands which are small could not bind so tightly in this slightly opened conformation. The volume and maximum extension of the cavities for chain A, which appeared the most interesting for docking, are shown in Figure 3C. Beyond intermediate number 10, the cavity is opened to the bulk solvent so its extension and volume are infinite according to the way these quantities are calculated. Conformations 4 and 10 together with the closed (1) and opened (49) crystallographic structures were selected as docking models for the virtual screening (Figure 4A).

### Virtual Screening, Candidate Ligand Selection and Testing

The 4 docking models were used in the two His 132 protonation states for the virtual screening of 31,000 compounds from the Chimiothèque Nationale (CN) and 630,000 compounds from the ChemDiv Chemical library (ChemDiv). The first two conformations led to the selection of small ligands, while conformations 10 and 49 selected compounds that were larger than PYC. This was reflected by the average molecular weight of the successfully docked compound in cavity 1, 4 and 10 (see Figure 3C). The average mass of compounds successfully docked in cavity 49 (382 Da = 191×2 Da) is more due to average mass of molecules in libraries than to the size of the cavity, which is unlimited. Noticeably however, conformation 4 selected slightly larger and heavier ligands than conformation 1. Following the procedure explained in Materials and Methods, 113 compounds were ordered from the ChemDiv and 37 from the CN and respectively 104 (92%) and 26 (70%) compounds were made available and tested for their ability to inhibit *Tc*PRAC.

Only two novel *Tc*PRAC inhibitors, both provided by the CN, were identified among these (Figure 4B). These compounds, both poorly reactive Michael acceptors - OxoPA (C_5_H_6_O_3_, MW 114,10, l_max_ = 6.07 Å, CSID:4515976, http://www.chemspider.com/Chemical-Structure.4515976.html) and its derivative BrOxoPA (C_5_H_5_O_3_Br, MW 193,00, l_max_ = 6.10 to 6.80 Å, [20] (Figure 4B) proved to be more potent than PYC (C_5_H_4_NO_2_, MW 110.09, l_max_ = 4,60 Å), in terms of *Tc*PRAC inhibition. Thus, based on a DAAOx test [34], equivalent concentrations of OxoPA and BrOxoPA inhibit more than 5 times the conversion of L- into D- proline catalyzed by *Tc*PRAC than that obtained with PYC (Table 2). Correspondingly, 3 µM of OxoPA and 2.5 µM of BrOxoPA are required for 50% inhibition (IC_50_) of *Tc*PRAC *in vitro*, as compared to 10 µM of PYC, as determined by polarimetry (Table 3).

**Table 2:** Two new compounds are more potent inhibitors of *Tc*PRAC than PYC.

Inhibitor	Inhibitor Concentration	D-Proline formed
(–)	(–)	5.0 µM
PYC	10 µM	2.4 µM (IC50)
OxoPA	10 µM	<0.3 µM
Br-OxoPA	10 µM	<0.3 µM

**Table 3:** Inhibition of *Tc*PRAC proline racemization measured by polarimetry.

Inhibitor	Inhibitor concentration	Racemization Inhibition
(–)	(–)	0.0%
PYC	10.0 µM	56.9%
OxoPA	3.0 µM	50.0%
Br-OxoPA	10.0 µM	81.3%
Br-OxoPA	2.5 µM	50.0%

**Figure 5:**
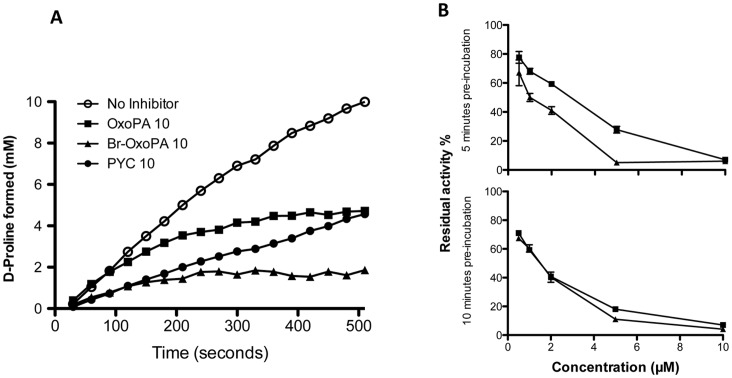
Kinetics of D-proline formation with time in the presence or absence of *Tc*PRAC inhibitors. (A) Concentrations of D-proline formed were determined by polarimetry in reaction assays containing 0.15 µM of *Tc*PRAC and 40 mM L proline (see Material and Methods). Optical rotation was measured every 10 s for 8 min, with 10 µM PYC competitive inhibitor (black circles), 10 µM OxoPA (black squares) and 10 µM Br-OxoPA (black triangles), or without inhibitor (white circles). (B) Percentage of residual activity after pre-incubation of the enzyme with different concentrations of OxoPA and BrOxoPA for 5 minutes (upper panel) and 10 minutes (lower panel).

**Figure 6:**
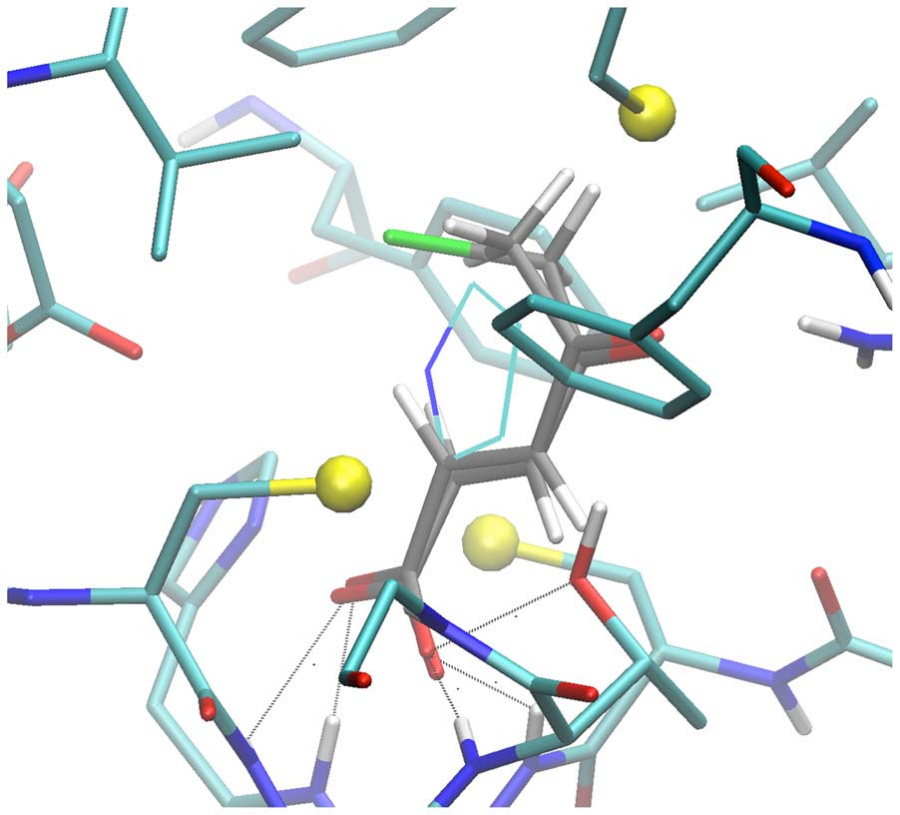
Close view of the docking pose of BrOxoPA and OxoPa in configuration 4. Enzyme residues are displayed in “licorice” with CPK colors and Carbons in cyan. Small spheres highlight sulfur atoms. Same representation with carbon in grey for inhibitor docking pose. Atoms forming bonds with the carboxylate of PYC in 1W62 structure are connected to corresponding pose inhibitor atoms. PYC molecule modeled by superposition of carboxylate and C2 atoms on that of BrOxoPA is displayed in lines, carbons in cyan.

Kinetic reaction assays using fixed amounts of dimeric PRAC (22 µg –0.24 µM final), 40 mM substrate and 10 µM of the identified inhibitors were performed to characterize their effect on enzymatic activity (Figure 5). PYC, the transition state analogue of proline, slows the racemization of L-proline (∼37% of original speed in these conditions). As it has been shown before PYC is a classical competitive and reversible *Tc*PRAC inhibitor and such as the reaction proceeds until racemic mixture is obtained [16]. The establishment of PYC inhibition is fast as shown by the slope of the curve at the origin and promptly reaches the steady-state (Figure 5A). In contrast, as compared with the curve obtained with PYC, *Tc*PRAC inhibition by OxoPA and BrOxoPA appears to be time-dependent since the initial velocity is not correlated with the global reduction of D-proline formation. For both OxoPA and BrOxoPA the curves of enzyme reaction progress are not linear with a final velocity close to zero. The rate of enzyme inactivation observed is faster for BrOxoPA than for OxoPA, since the plateau is reached at 250 seconds for BrOxoPA when it requires 350 seconds for OxoPA. The rate of inactivation reaction and the slope of the inhibition curves obtained with these two compounds suggest that these molecules present distinct affinities and/or reactivity for the catalytic site of the enzyme. In addition, excess of substrate (300 mM of L- proline, data not shown) induced observable protection from inactivation by these two compounds, which suggested that the inactivation process took place in the catalytic pocket. To further characterize the mode of action of this two molecules, the enzyme was pre-incubated (concentration 22 µg − 3,6 µM) with different concentration of inhibitor, for 5 or 10 min to determine inhibition dependence to inhibitor concentration and pre-incubation time. Then, the reaction mixture was diluted 15 fold and residual activity was determined in an excess of substrate (300 mM of L- proline). As can be seen in Figure 5B, BrOxoPA inactivation is faster than the one observed for OxoPA and in both cases the rates of inactivation are dependent on the inactivator concentration and time, suggesting a classical irreversible reaction. Besides, irrespective of the fact that BrOxoPA and OxoPA bind to the catalytic site and cause rapid or slow inactivation of the enzyme both compounds are very strong and irreversible *Tc*PRAC inhibitors. The presence in OxoPA and BrOxoPA of a carboxylic acid function like in Proline and PYC is an important element of recognition of these compounds. Furthermore, the sp2 C-2 carbon of both OxoPA and BrOxoPA, matching the inverted C_α_ of the proline and the sp2 C-2 of PYC, is an electrophilic center that can be covalently bounded with a nucleophylic amino acid such as a cysteine residue. One can suppose that the carboxylic acid moiety holds the electrophilic C-2 reacting group at a mutual distance of the two cysteine residues (Cys-130 and Cys-270 that are involved in the proline isomerisation), which highly favors the reaction. In fact, in the docking leading to the selection of the OxoPA and BrOxoPA, their carboxylate group was close to the moieties making hydrogen bonds to the PYC carboxylate in the X-ray structure. Noticeably, the FlexX program, which is based on fragmentation-reassembly of the ligand, rebuilt ligands in poses with a cis C3–C4 bond (Figure 6). Those shorter conformers allowed positioning of the C2 atom close to the middle of the pair of catalytic sulfur atoms with an occupied volume slightly larger than that of the modeled PYC (drawn in lines; l_max_ = 5.27 and 5.38). Although docking poses are usually poorly reliable, this shows that those molecules could make highly specific interactions with limited adjustment. Finally, we pursue enzyme crystallization with OxoPA and BrOxoPA to elucidate whether they are covalently attached to target catalytic residues of *Tc*PRAC (experiment in progress).

### Mammalian Cell Invasion by the Parasite is Hampered by New Inhibitors of *Tc*PRAC

**Figure 7:**
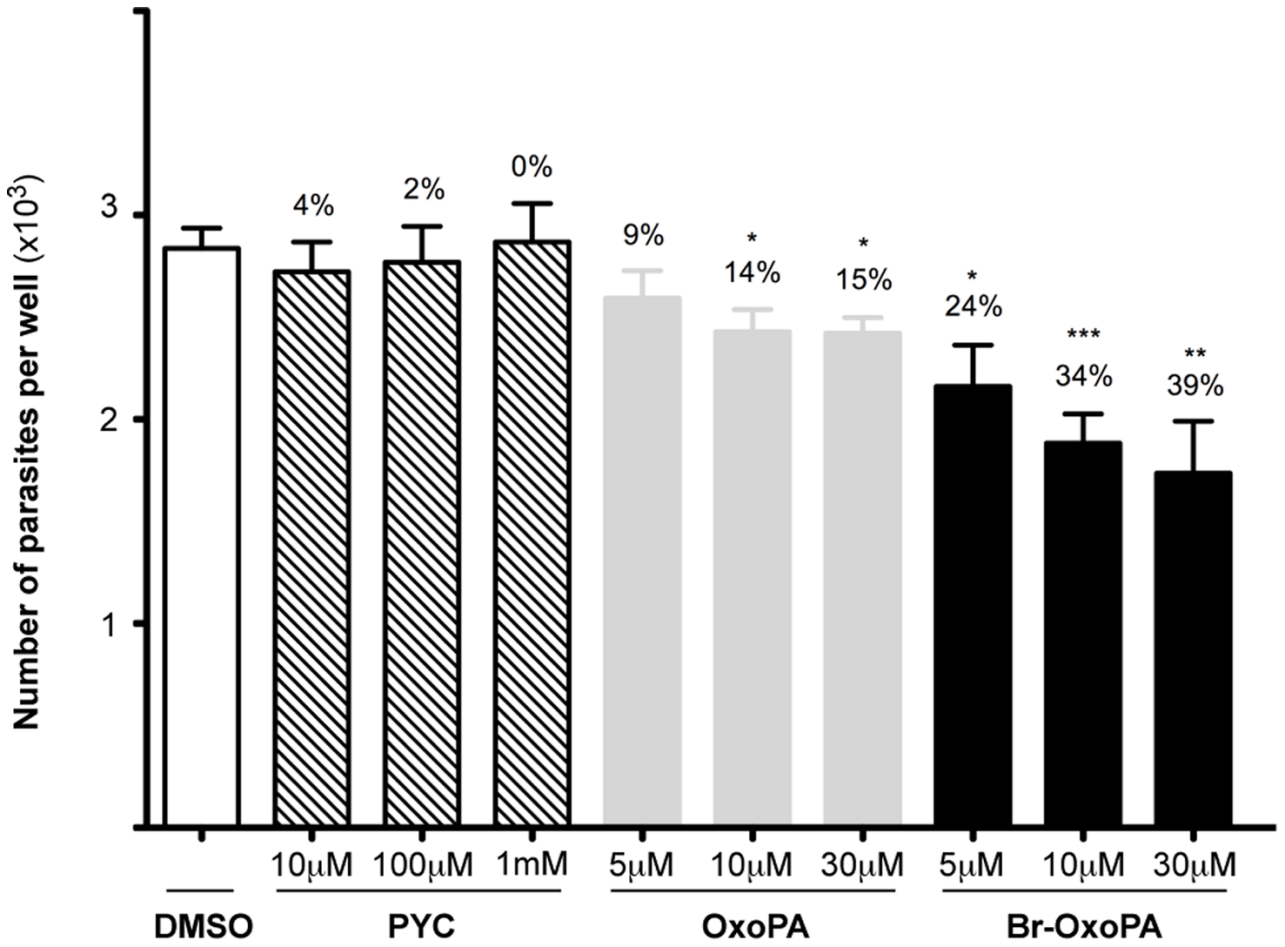
Effects of *Tc*PRAC inhibition by OxoPA and BrOxoPA on parasite interaction with host cells in vitro. Vero cells cultures were infected for 17 h at 37°C with cultured trypomastigotes at a 10∶1 parasite/cell ratio then washed and incubated for 17 h with 0, 100 and 1000 µM of PYC, or 0, 10 and 30 µM OxoPA or Br-OxoPA in fresh medium. Total parasite numbers/culture were estimated by capture ELISA.

To verify the potential of these new PRAC inhibitors as lead compounds in drug design strategies, we compared their effects on cultures of host Vero cells infected or not with *T. cruzi*, with that of PYC. Our results showed that when increasing PYC concentrations (3, 10 µM, 100 µM, 1000 µM) are added concomitantly with trypomastigotes and host cells *in vitro*, a clear dose-dependent effect was noted resulting in fewer infected host cells, and lower mean numbers of parasites per infected host cell (Table 4). In comparison, lower doses of OxoPA or Br-OxoPA (100- and 30-fold, respectively) were necessary to obtain the same endocytic index (EI) as that observed with culture cells treated with PYC. For instance, while control samples treated with comparable amounts of DMSO did not show any particular alterations, equivalent EI were observed for 1000 µM PYC and 10 µM OxoPA or 30 µM of Br-OxoPA. It is noteworthy that neither OxoPA nor Br-OxoPA considerably affected the viability of non infected Vero cells, which maintained adhesion to the support at the tested concentrations (3, 10 and 30 µM). To confirm the effect of the inhibitors in the initial steps of the host-parasite interaction (parasite uptake), cultures were infected for 17 h at 37°C at a 10∶ 1 parasite/cell ratio with or without increasing doses of freshly prepared dilutions (5, 10, 30 µM) of OxoPA, Br-OxoPA, or (10, 100, 1000 µM) of PYC, previously dissolved in DMSO and further analyzed by capture ELISA. Although no particular differences were observed in cultures treated with PYC diluted in DMSO, as in Table 4 a dose dependent inhibition of cellular infection was induced by both inhibitors, most particularly with Br-OxoPA. To determine whether the treatment of these cultures with the new compounds would indeed affect intracellular *Tc*PRAC and as such interfere with parasite intracellular fate cells were then washed after parasite uptake and the same ligand concentrations as above (10 µM − 30 µM) were added to the cultures in fresh medium. The degree of infection was determined over 48 hours by capture ELISA. As expected, the number of parasites per well decreased in a dose-dependent manner (Figure 7). *In vitro* and *in vivo* experiments using mutant parasites that carry the luciferase gene are now in progress to support those data. In conclusion, these experiments indicate that *Tc*PRAC inhibition by OxoPA and Br-OxoPA affects both cellular infection by *T. cruzi* and parasite development in tissues.

**Table 4:** New inhibitors induce a decrease of cellular uptake of *T. cruzi*.

		Inhibitor concentration (µM)					
	Compound	0	3	10	30	100	1000
**Endocytic index (EI)**	PYC	45	*nd*	44	*nd*	**30**	**25**
	OxoPA	57	42	**23**	**21**	*nd*	*nd*
	Br-OxoPA	57	78	42	29	*nd*	*nd*

## Discussion

The data presented herein substantiate previous findings showing that PRAC plays a role in both *T. cruzi* development and infectivity and further support its use as a original target for the development of a new chemotherapy against Chagas’ disease [7], [15], [39], [40].

Modeling the protein functional trans-conformation by molecular mechanics and using the plausible intermediate conformers thus built for virtual screening happened to be a fruitful approach since OxoPA and Br-OxoPA would not have been selected if the screening had been performed on the crystallographic structure alone. This puts forward and substantiates the idea that targeting putative functional intermediate structures could open new doors in rational drug design and gives access to molecules with novel mode of action [25], [41]. However, intermediate structures build by modeling methods remain hypothetical and many examples such as the work presented here and others [25], [41] will be necessary to fully establish this type of approach. Other methods to explore structural transition such as umbrella sampling and other free energy based methods could be used. However, they require a previous knowledge of a relevant geometrical description of the mechanism (e.g. by collective variables), and the transition path model is given as an ensemble of millions intermediate structures, which may be difficult to exploit in a virtual screening approach. POE (see Materials & Methods) almost unavoidably requires use of an implicit solvent model to ensure immediate and room temperature equilibration of the latter in the context of optimization. ACE2 is a highly robust generalized Born implicit model. It is approximately 10 times slower that fast implicit models like sigmoidal electrostatic models or EEF1 implemented in CHARMM [42], but it is also about 10 times faster than conventional explicit water simulations. It has proved to be very effective with POE since optimization performs better than with cruder models with better convergence properties and building of better conformations, which is important for docking [25].

Interestingly, the keto-acids compounds identified in this work and acting as Michael acceptors like in many drugs actually in development [43], [44], differ considerably from PYC and proline residues. Noticeably, many drugs appear to be covalent inhibitors and up to 35% of the current drug targets could be covalently inhibited although the discovery programs were not seeking such mechanism [45]. The suitability of developing covalent inhibitors appears to be favorably reconsidered [46]. Noteworthy, the current inhibitors would have been discarded if a classical “drug-like” filter had been applied. It had been decided not to apply such classical filters due to small size of the targeted site and the previous unsuccessful attempts to identify *Tc*PRAC inhibitor, substantiating the idea that no chances to identify an inhibitor for this unconventional site should be put aside. Additionally, it would have been difficult to identify those compounds by classical medical chemistry or QSAR methods. Another interesting contextual consideration to have, bearing in mind the suitability of covalent drugs, is that virtual screening methods are mostly conceived for non covalent interactions. In the current work, a slight leap helped us in finding the current inhibitors. The detailed topology of the molecule described in the virtual libraries did not clearly specified the conjugated nature of the molecule and the program most likely fragmented the molecule between C3 and C4 before reconstruction in the cis conformation allowing closer superposition with PYC structure (e.g. Figure 6). We maintained this compound in the order list, considering that some spontaneous isomerization could occur. This reconstruction together with the larger volume offered in conformation 4 probably give sufficient flexibility to accommodate the current compounds, hence effectively enlarging the chemical search space.

OxoPA and Br-OxoPA not only inhibit *Tc*PRAC biochemically but also have effects both on cellular infection by *T. cruzi* and on parasite intracellular development. This shows their efficacy against essential parasite processes and consequently supports their therapeutic potential. These compounds have also been shown to reach their intracellular target, clearly demonstrating their diffusion through both cell and parasite membranes. Finally, no significant, detectable toxicity against mammalian cells was observed *in vitro*. Altogether, these properties indicate that OxoPA and Br-OxoPA are promising leads for the development of new therapies against the chronic phases of the disease.

Ongoing experiments aim to design and synthesize even more effective OxoPA and Br-OxoPA derivatives with improved affinity and specificity for *Tc*PRAC and appropriate pharmacokinetic properties. One line of improvement is to synthesize compounds with lower electrophilic reactivity to decrease potential sources of toxicity in a complex physiological environment. From these two promising inhibitors, a set of candidate structures can be synthesized that will improve both the non-covalent binding affinity and the positioning of the electrophilic site with respect to the nucleophylic partner (Cys residues) in the catalytic site to optimize potency and selectivity. The nature of the electrophilic moiety will be investigated. Ideally, this electrophile should be poorly reactive with nucleophiles under physiological conditions but upon appropriate orientation should selectively react in the catalytic site. A key step in this process is to refine the crystallographic structures of the enzyme complexed with these inhibitors to better support medicinal chemistry approach. Such studies would strengthen the structural hypotheses used to identify these compounds and provide more robust data for the lead optimization process. Since we have recently shown that important pathogens of the *T. cruzi* family [47] and also nosocomial bacteria produce functional proline racemases [48], our data suggest that these chemicals may also be useful in other diseases where proline racemases are vital.

## Supporting Information

Table S1 **Pyrazole-bearing analogues of PYC were synthesized by conventional and specific procedures, as described in the Material and Methods.** Compound abbreviations used throughout the manuscript text are shown in parenthesis.
(ZIP)

Table S2 **Initial attempts to identify inhibitors based on a pharmacophoric approach under** ***Tc*** **PRAC crystallographic structure insight.** Six pharmacophoric models were built and tested (software Catalyst 4.11 (1252), Acceryls Software, Inc) with by then known weak inhibitors: PYC, PZC, Cl-PZC, Br-PZC and non-inhibitor but also hydro soluble related compounds built by medicinal chemistry (see Materials and Methods). The selectivity of the pharmacophore and the three-dimensional analysis in terms of size and volume of the *Tc*PRAC catalytic site cavity (1W61 and 1W62 PDB codes, publicly available) guided the choice. The binding of PYC to *Tc*PRAC is maintained by a network of hydrophobic and hydrophylic interactions, as it has been reported in details by crystallographic studies [18]. An initial pharmacophore was automatically generated based on PYC using the “Hypogen” procedure. It displayed 1 hydrogen bond donor, 2 hydrogen bond acceptors and 1 aromatic ring features. This pharmacophore was refined incrementally by the study of the *Tc*PRAC catalytic site structure to avoid numbers of false positive/negatives in the known inhibitor/non-inhibitor set. Hence, the analysis of the best inhibitor, PYC, showed that Phe-290 benzene ring forming one of the binding pocket wall lead to steric constraints that could disallow the binding of larger molecules and impose hydrophobicity restrictions. Thus, the overlay of the pharmacophore 3D-coordinates on the PYC/*Tc*PRAC complex 3D structure guided the definition and constraints of the exclusion volume and shape added to the initial pharmacophore. More than 2 200 K molecules from 7 chemical compound libraries were considered. Respecting the volume constraints, a molecular mass filter (MW <350) was applied and 361093 molecules were thus selected from 3 major providers (Asinex, LifeChemical and ChemDiv) themselves selected according to the number of filtered compounds and availability criteria. 3D conformations of the selected compounds were generated using the “FAST” algorithm of DiscoveryStudio. Two other filters, acquous solubility features (−4<logSw<0) and refined pharmacophore, were applied to those molecules and allowed the selection of molecules similar to PYC with relaxed shape restraints (CatSearch). Then, compounds were filtered for the Lipinski rule of 5 (well represented in the selected libraries) in order to extract drug-like molecules. Finally, compounds were clustered hierarchically (FCFP fingerprint) to allow classification at different level and define diversity subsets of to desired sizes. A first experimental screening campaign has applied to the 1000 diversity subset of the “ChemDiv, Inc” chemical library with no success in finding an inhibitory activity for *Tc*PRAC, as assayed by polarimetry and by D-amino acid oxidase- based method. Another set of 374 best-fit compounds was selected among the proposed options and compounds were purchased, 330 received, and as previously, no *Tc*PRAC inhibitor was identified among them (*Minoprio, P. and Afshar, M., unpublished*).
(ZIP)
