# Supplementary material for: Combined Approaches for Drug Design Points the Way to Novel Proline Racemase Inhibitor Candidates to Fight Chagas’ Disease
Source: PLoS One. 2013 Apr 16;8(4):e60955. doi: 10.1371/journal.pone.0060955 (PMC3628851; doi:10.1371/journal.pone.0060955)
Supplement: Table S2 — Initial attempts to identify inhibitors based on a pharmacophoric approach under Tc PRAC crystallographic structure insight. Six pharmacophoric models were built and tested (software Catalyst 4.11 (1252), Acceryls Software, Inc) with by then known weak inhibitors: PYC, PZC, Cl-PZC, Br-PZC and non-inhibitor but also hydro soluble related compounds built by medicinal chemistry (see Materials and Methods). The selectivity of the pharmacophore and the three-dimensional analysis in terms of size and volume of the TcPRAC catalytic site cavity (1W61 and 1W62 PDB codes, publicly available) guided the choice. The binding of PYC to TcPRAC is maintained by a network of hydrophobic and hydrophylic interactions, as it has been reported in details by crystallographic studies [18]. An initial pharmacophore was automatically generated based on PYC using the “Hypogen” procedure. It displayed 1 hydrogen bond donor, 2 hydrogen bond acceptors and 1 aromatic ring features. This pharmacophore was refined incrementally by the study of the TcPRAC catalytic site structure to avoid numbers of false positive/negatives in the known inhibitor/non-inhibitor set. Hence, the analysis of the best inhibitor, PYC, showed that Phe-290 benzene ring forming one of the binding pocket wall lead to steric constraints that could disallow the binding of larger molecules and impose hydrophobicity restrictions. Thus, the overlay of the pharmacophore 3D-coordinates on the PYC/TcPRAC complex 3D structure guided the definition and constraints of the exclusion volume and shape added to the initial pharmacophore. More than 2 200 K molecules from 7 chemical compound libraries were considered. Respecting the volume constraints, a molecular mass filter (MW <350) was applied and 361093 molecules were thus selected from 3 major providers (Asinex, LifeChemical and ChemDiv) themselves selected according to the number of filtered compounds and availability criteria. 3D conformations of the selected compounds were generated [file pone.0060955.s002.zip › Table S2.docx]

**Table S2 : Data base selection and filtering**

| **Step 1 : Database** | **# compound** | **Size filter** | **Filtered** | **Selected** |
| --- | --- | --- | --- | --- |
| **ChemDiv, Inc** | 682 329 | MW < 350 | 173 037 | **173 037** |
| **AsinexGold Collection** | 233 795 | MW < 350 | 107 100 | **107 100** |
| ChemStar2006 | 60 040 | MW < 350 | 22 865 |  |
| Maybridge | 56 842 | MW < 350 | 33 870 |  |
| **Life_Chemical_stock** | 296 076 | MW < 350 | 80 956 | **80 956** |
| InterBioScreen_nc | 42 227 | MW < 350 | 12 194 |  |
| InterBioScreen_nc1 | 197 750 | MW < 350 | 63 722 |  |
| InterBioScreen_nc2 | 176 263 | MW < 350 | 46 359 |  |
| Chembridge_EXP | 454 964 | MW < 350 | 218 475 |  |
| Chembridge_MW | 30 000 | MW < 350 | 18703 |  |
| **Total** | **2 230 286** | **MW < 350** | **777 281** | **361 093** |
| **Step 2 : Filters** | **ChemDiv** | **AsinexGold** | **Life_Chemical** | **Total** |
| # compounds | 682 329 | 233 795 | 296 076 | 1 212 200 |
| MW≤350 | 173 037 | 107 100 | 80 956 | 361 093 |
| CatSearch | 12 029 | 7 787 | 6 756 | 26 572 |
| logSw [-4;0] | 7 351 | 4 703 | 3 387 | 15 441 |
| Linpinski "rule of 5" | 7 323 | 4 687 | 3 371 | 15 381 |
| 6% diverse subset | 441 | 282 | 203 | 926 |
| 3% diverse subset | 220 | 141 | 102 | 463 |
| 1% diverse subset | 73 | 47 | 33 | 153 |
| 1000 diverse subset (≈13.6%) | 1000 |  |  | 1000 |
| 100 bestFit per Cluster | 100 | 100 | 100 | 300 |
| bestFit per Cluster+ SIMfilter | 237 | 137 |  | 374 |
| shape constraint | 9 | 10 | 5 | 24 |
