# Supplementary material for: Combined Approaches for Drug Design Points the Way to Novel Proline Racemase Inhibitor Candidates to Fight Chagas’ Disease
Source: PLoS One. 2013 Apr 16;8(4):e60955. doi: 10.1371/journal.pone.0060955 (PMC3628851; doi:10.1371/journal.pone.0060955)
Supplement: Table S1 — Pyrazole-bearing analogues of PYC were synthesized by conventional and specific procedures, as described in the Material and Methods. Compound abbreviations used throughout the manuscript text are shown in parenthesis. (ZIP) [file pone.0060955.s001.zip › Table S1.doc]

**Table S1 : PYC and synthesized pyrazole analogues**

| **Compound (Abbreviation)** | **Formula** |
| --- | --- |
| Pyrrole-2-carboxylic acid (PYC) |  |
| Pyrazole-3-carboxylic acid (PZC) |  |
| 4-Chloro-pyrazole-3-carboxylic acid (Cl-PZC) |  |
| 4-Bromo-pyrazole-3-carboxylic acid (Br-PZC) |  |
| 4-Ethyl-pyrazole-3-carboxylic acid (Et-PZC) |  |
| 4-Chloro-5-methyl-pyrazole-3-carboxylic acid (M-Cl-PZC) |  |
